# Evaluation of endoscopic variceal band ligation sessions in obliteration of esophageal varices

**DOI:** 10.12669/pjms.36.2.1144

**Published:** 2020

**Authors:** Haris Alvi, Bader Faiyaz Zuberi, Tazeen Rasheed, Muhammad Asad Ibrahim

**Affiliations:** 1 Haris Alvi, Department of Medicine, Dow University of Health Sciences, Karachi, Pakistan; 2 Bader Faiyaz Zuberi, Department of Medicine, Dow University of Health Sciences, Karachi, Pakistan; 3 Tazeen Rasheed, Department of Medicine, Dow University of Health Sciences, Karachi, Pakistan; 4 Muhammad Asad Ibrahim, Department of Medicine, Dow University of Health Sciences, Karachi, Pakistan

**Keywords:** Endoscopic variceal band ligation, Esophageal varices

## Abstract

**Objective:**

To determine number of sessions of Endoscopic variceal band ligation required to obliterate the esophageal varices.

**Methods:**

This study was conducted at Civil Hospital Karachi between June 2018 to April 2019. All patients undergoing endoscopic Variceal Band Ligation (EVBL) were inducted. Number of sessions of band ligation required to obliterate the varices were recorded. Number of EVBL sessions were correlated with Child-Pugh’s Score and etiology of CLD by χ^2^ test, while it was compared with duration of Chronic Liver Disease (CLD) by One-way ANOVA test.

**Results:**

One hundred ninety-two patients fulfilling selection criteria were admitted after informed consent. These included 101 (52.6%) males and 91 (47.4%) females. Most common cause of cirrhosis was HCV (66.7%) in our patients. Most of the patients were in Child Class-B (71.9%). Majority of patients (52.6%) underwent 3 sessions of EVBL while 68 (35.4%) underwent 4 sessions of EVBL. Duration of CLD was analyzed with number of sessions of EVBL by One-Way ANOVA test and it showed significant more sessions of EVBL were done with longer duration of CLD (p <0.001).

**Conclusion:**

Most patients underwent 3-4 sessions of EVBL for obliteration of varices. Number of EVBL sessions increased significantly with duration of disease.

## INTRODUCTION

Variceal bleeding is a serious life-threatening complication of portal hypertension.^1^ Portal hypertension is part of a dynamic process triggered by chronic liver disease. There are many etiologies of chronic liver disease, the most common being chronic viral hepatitis in our part of the world and alcohol in the western world.^2^-^5^ In cirrhosis there is an increase in the intrahepatic vascular resistance as a result of fibrosis and a rise in the vascular tone of the hepatic microcirculation.^6^ The rise in portal pressure causes collateral circulation to develop as a result portal blood is diverted into the systemic circulation. In decompensated cirrhosis portal hypertension is a serious problem and it may present as ascites, splenomegaly and esophageal varices.^3^ Hepatitis C (HCV) is the commonest cause of cirrhosis in our part of the world.^5^ About 50% of the cirrhotic patients develop esophageal varices and among them 35% will die in their first episode.^7^ There are different grades of varices and they may be classified as small, medium and large varices.^8^ Endoscopic Variceal Band Ligation (EVBL) is effective mode to treatment not only to arrest active bleed, but in eradication of varices. Multiple sessions of EBVL are required to obliterate the varices.^9^

Successful obliteration of varices decreases the likelihood of variceal bleed and thus decreases morbidity and mortality.^5^ Obliteration of varices is an important milestone in management of cirrhosis. We searched Medline via PubMed and local index PakMediNet for publications on this topic which showed very scanty data worldwide and none from Pakistan. There is lack of information on this topic and need to conduct study on this aspect of liver disease.

Our objective was to determine the number of sessions of EVBL required to eradicate the esophageal varices in cirrhotic patients. This study will help in better understanding of this disease in our settings and better formulation of management and prediction plans in our population.

## METHODS

This study was conducted at Civil Hospital Karachi between June 2018 to April 2019. Patients satisfying inclusion/exclusion criteria were included after informed written consent. Approval (IRB-1030/DUHS/Approval/2018/80 dated May 31st, 2018) was taken from the Institutional Review Board of Dow University of Health Sciences. All patients undergoing endoscopic Variceal Band Ligation (EVBL) were inducted. Number of sessions of band ligation required to obliterate the varices and number of bands applied per session were recorded. Patients having gastric varices, patients with a history of sclerotherapy or previous band ligation, rebleed after EVBL, patients with hepatic encephalopathy, patients with a platelet count of <50000/mm^3^, INR of >1.8 were excluded. EVBL sessions were performed by faculty who have certified training in doing EVBL and related endoscopic procedures. All patients were started with Tab Carvidalol 6.25 mg bid next day of EVBL unless there was any contra-indication. Those with contraindications were excluded from analysis but continued treatment and further sessions. Varices were defined as obliterated if there were no varices on visual inspection on endoscopy or were too small for EVBL.

All the patients were evaluated by one of the authors about clinical history and examination. The blood tests performed were Blood CP, PT/INR. All EVBL were performed in a single endoscopy unit using Olympus video scope GIF 180 by experienced gastroenterologists. First vitals were checked, and patients were sedated with midazolam intravenously according to the weight of the patient. A screening endoscopy was done and if medium to large size esophageal varices were present then bands were applied by using multi-load ligature device. Patient were observed for an hour after the procedure and discharged with clear written advice. Patients were advised to revisit after 3 weeks with fresh complete blood count and prothrombin time for next session of endoscopy and if needed then band ligation.

Grading of esophageal varices is given as under which is based on approximate size of standard open biopsy forceps is equal to 5 mm:^6^

• Small: < 5 mm

• Large: > 5 mm

### Child Turcotte Pugh (CTP)

In this classification two clinical sign, i.e., ascites and hepatic encephalopathy and three laboratory parameters, i.e., bilirubin, serum albumin and INR are included. They are classified according to the points system and is designated as class A (5-6 points), class B (7-9 points) and class C (10-15 points).

### Sample Size

Using WHO calculator Sample size was calculated by taking prevalence as 22.1% for two sessions, with margin of error 8%. The power of this analysis is 95% and p-value of 0.05. The sample size was estimated to be 104 patients. Sample technique was non-probability consecutive sampling.

### Data Analysis Procedure

Data was entered in SPSS version 25.0. Frequencies and percentages for gender, Child-Pugh’s Class, etiology and number of sessions of EVBL were done. Mean ±SD were reported for age and duration of CLD. Mean age was compared with gender by student’s t-test. Number of EVBL sessions were correlated with Child-Pugh’s Score and etiology of CLD by χ^2^ test, while it was compared with duration of CLD by One-way ANOVA test. Level of significance was set at ≤0.05.

## RESULTS

One hundred ninety-two patients fulfilling selection criteria were admitted after informed consent. These included 101 (52.6%) males and 91 (47.4%) females. Mean age was 47.4 ±8.2 years. Mean age of males was 47.4 ±0.9 years while that of females was 47.7 ±7.5 years. There was no statistical difference in age among gender (*p* = 0.811; 95% CI -2.63 to 2.06). Most common cause of cirrhosis was HCV (66.7%) in our patients. Most of the patients were in Child Class-B (71.9%). Majority of patients (52.6%) underwent 3 sessions of EVBL, while 68 (35.4%) underwent 4 sessions of EVBL. Details are given in Table-I. Number of sessions of EVBL was tested for significance with Child-Pugh’s Class and Etiology of CLD by using *χ^2^*-test. Test reveal 8 (53.3%) cells had expected value of <5 so Likelihood Ratio was calculated, and it showed significantly higher number of EVBL done in Child Class-B (*p* = 0.035). Similarly, significantly higher frequencies of HCV were found as etiology in our study (*p* = 0.013). Number of sessions were compared with duration of disease and age by One Way ANOVA. It showed significant more sessions of EVBL were done with longer duration of CLD (*p* <0.001) while no significant difference was found with age (*p* = 0.967). Details are given in Table-II. Sub-analysis of ANOVA between sessions of EVBL was done using Games-Howell Method. It showed following results in between different sessions of EVBL:

**Table-I:** Etiology, Child-Pugh’s score & number of EVBL sessions according to gender.

		Sex of Patient				Total

		Male		Female		

		Count	%	Count	%	Count (%)
Etiology of CLD	HCV	64	63.4%	64	70.3%	128 (66.7%)
	HBV	19	18.8%	25	27.5%	44 (22.9%)
	Alcohol	14	13.9%	0	0.0%	14 (7.3%)
	Wilson’s disease	4	4.0%	2	2.2%	6 (3.1%)
Child-Pugh’s Score	A	7	6.9%	5	5.5%	12 (6.3%)
	B	80	79.2%	58	63.7%	138 (71.9%)
	C	14	13.9%	28	30.8%	42 (21.9%)
Number of Sessions of EVBL	two	6	5.9%	4	4.4%	10 (5.2%)
	three	56	55.4%	45	49.5%	101 (52.6%)
	four	36	35.6%	32	35.2%	68 (35.4%)
	five	3	3.0%	7	7.7%	10 (5.2%)
	six	0	0.0%	3	3.3%	3 (1.6%)

**Table-II:** Cross Tabulation of EVBL sessions with CP score, Etiology & duration of CLD with statistical analysis.

		Number of Sessions of EVBL					p-value

		Two	Three	Four	Five	Six	
Child-Pugh’s Score	A	0	8	2	2	0	0.035*
	B	10	74	48	3	3	
	C	0	19	18	5	0	
Etiology of CLD	HCV	10	62	43	10	3	0.013*
	HBV	0	25	19	0	0	
	Alcohol	0	10	4	0	0	
	Wilson’s disease	0	4	2	0	0
Mean Duration of Disease (years)		3.4	4.48	6.76	8.0	9.67	<0.001*
Mean Age (years)		46.6	47.5	47.4	49.0	47.5	0.967*

Significance Level ≤0.05, χ^2^ test with Likelihood Ratio, χ^2^ test with Likelihood Ratio, One-Way ANOVA, One Way ANOVA.

• Two/Three: Non-significant

• Three/Four: Significant

• Four/Five: Non-significant

• Five/Six: Non-significant

Details are given in Table-III.

**Table-III:** Multi-level comparison of duration of CLD with EVBL sessions by ANOVA Games-Howell Method.

Multiple Comparisons						

Dependent Variable: Duration of Disease						

Games-Howell						

(I) Number of Sessions of EVBL	(J) Number of Sessions of EVBL	Mean Difference (I-J)	Std. Error	Sig.	95% Confidence Interval	

					Lower Bound	Upper Bound
Two	Three	-1.07525	0.64288	0.478	-3.0554	0.9049
	Four	-3.36471*	0.64949	0.001	-5.3569	-1.3725
	Five	-4.60000*	0.73333	0.000	-6.8245	-2.3755
	Six	-6.26667*	0.87178	0.004	-9.6947	-2.8386
Three	Two	1.07525	0.64288	0.478	-.9049	3.0554
	Four	-2.28946*	0.45169	0.000	-3.5360	-1.0430
	Five	-3.52475*	0.56565	0.000	-5.2319	-1.8176
	Six	-5.19142*	0.73633	0.023	-9.1224	-1.2604
Four	Two	3.36471*	0.64949	0.001	1.3725	5.3569
	Three	2.28946*	0.45169	0.000	1.0430	3.5360
	Five	-1.23529	0.57316	0.238	-2.9582	.4876
	Six	-2.90196	0.74211	0.107	-6.7783	.9744
Five	Two	4.60000*	0.73333	0.000	2.3755	6.8245
	Three	3.52475*	0.56565	0.000	1.8176	5.2319
	Four	1.23529	0.57316	0.238	-.4876	2.9582
	Six	-1.66667	0.81650	0.379	-5.1823	1.8489
Six	Two	6.26667*	0.87178	0.004	2.8386	9.6947
	Three	5.19142*	0.73633	0.023	1.2604	9.1224
	Four	2.90196	0.74211	0.107	-0.9744	6.7783
	Five	1.66667	0.81650	0.379	-1.8489	5.1823

* . The mean difference is significant at the 0.05 level.

## DISCUSSION

Cirrhosis is a leading cause of mortality and morbidity worldwide. Bleeding from esophageal varices is one of the most serious complications of cirrhosis having a high mortality.^10^ Patients who survive a first episode of variceal bleeding have a 60% risk of experiencing rebleed. Therefore treatment to prevent first episode of variceal haemorrhage is of utmost importance and all patients surviving a variceal bleed must receive treatment to prevent rebleeding.^1^ EVBL is the mainstay in the management of esophageal varices.

In our study we aimed to find out the number of sessions of EVBL required to obliterate esophageal varices. Successful eradication was taken as disappearance of varices or presence of small varices which could not be ligated. In our study majority of patients were from Child-Pugh Class-B and the majority of patients required 3-4 banding sessions to obliterate the esophageal varices in 93.2% of our patients. Our findings were supported by a study by Khattak AB in which majority of patients achieved complete eradication after 3 sessions of band ligation.^11^ In a study by Lahbabi M et al. it was reported that obliteration of the varices was achieved in 89.6% of patients with 3±1.99 sessions of band ligation.^12^ There are variable views regarding schedule of EVBL sessions. We followed flexible 2-4 weeks’ interval in EVBL sessions. While some favor’s weekly versus bi-weekly schedule and others favor bi-monthly schedule.^13^,^14^ Pain and post banding ulcers were found more frequently in short interval sessions protocols but late obliteration of varices occurred with long interval protocols. Shrestha B et al. in their study reported that in the majority of their patients, varices were eradicated after 2 sessions of band ligation and 24.1% of patients required just 1 session of EVBL whereas in our study in none of the patients the varices were obliterated after 1 session.^10^

Another significant finding that came out from out study was that the number of sessions were significantly increased with longer duration of cirrhosis. Its explanation could be that with longer duration of CLD larger varices were formed that took more sessions to obliterate.

In our study the most common etiological cause for cirrhosis was HCV. In a recent article by Kim D et al. the authors reported significant decrease in HCV related mortality during the period 2014 to 2106, this correlates to the era of directly acting antivirals.^5^ We have not witnessed such a phenomena yet, but we hope to see it in near future.

### Limitation of the study

The limitation of our study was that ours was a single center study and we do not have the follow up data of our patients after successful eradication of varices regarding rebleeding and recurrence of varices.

## CONCLUSION

Our study showed that majority of varices were eradicated with 3-4 sessions of EVBL and longer duration of CLD required more sessions.

### Author`s Contribution:

**HA:** conceived and designed the study and edited manuscript, is responsible for integrity of research.

**BFZ:** did statistical analysis and gave final approval of manuscript.

**TR:** wrote and corrected manuscript.

**MAI:** collected data, wrote draft manuscript.
